# Extracts and Gleanings

**Published:** 1872-12

**Authors:** 


### PART II.

### Abridged Extracts and Gleanings from our Exchanges

*MULTUM IN PARVO.*

"Use of Nux Vomica in Certain Neuroses of Organic Life.—M. Brugnoli has employed nux vomica successfully in the nervous movements of pregnancy, gastralgia, dyspepsia, hypochondriasis, nervous palpitations of the heart, nervous and periodic cough, asthma, and finally, in albuminuria. This remedy acts either on the pneumogastric, or on the great sympathetic, or on the spinal cord. He records the case of a lady affected with a severe cough recurring every evening, and lasting throughout the night, who was cured in two days by the use of the nux vomica. Another patient was affected every evening with violent cough, accompanied by catarrhal expectoration, and was also cured in two days by the use of the alcoholic extract of nux vomica, mixed with the extract of gentian. Cough may always be allayed by this means, whether it be caused by bronchitis, by pneumonia, by pulmonary phthisis, or by emphysema. It proves a remedy also in cases of cardiac pulsations, and in irregular or too frequent action of the heart. In albuminuria, M. Brugnoli has retarded its progress to some extent, especially in cases of scarlatinal albuminuria."—*Journal de Medicine.*

"This medicine, however, should be administered with judgment, and never given in cases depending on inflammation or organic lesion of the brain or spinal marrow, until after the removal of the primary affection."—*U. S. D.*

"Oxalate of Potash in Peritonitis.—Two cases of peritonitis with purulent effusion, the pus discharging itself by the umbilicus, have been reported this year to the Medico-Chirurgical Society of Liege, as resulting successful. Other cases have also been reported, in which small doses of oxalate of potash in mucilage were given every hour, with the best results, the patients being perfectly and speedily restored to health."

A Positive Sign of Death.—In 1870 the Academy of Sciences of Paris offered a price of twenty thousand francs for some simple and positive sign of death, one which can be applied at any time by non-medical persons, requiring no instruments, and unmistakable in its indications.

Of course, a number have been spoken of. The latest, and perhaps the best, is that suggested by Dr. Hugo Magnus, of Breslau, in one of the latest numbers of Virchow's Archiv. It is simple, physiological and conclusive. Everybody knows that when the circulation positively ceases, the man is dead. No matter how profound the coma or trance, no matter how death-like the lethargy, some circulation must continue, be it ever so sluggishly. When it stops once, resuscitation is impossible.

All that one has to do, therefore, is to tie a string firmly around the finger of the supposed corpse. If there is the least spark of life left—that is, if the blood circulates at all, the whole finger, from the string to the tip, will gradually turn a bluish red, from the engorgement of the veins. Nothing else, no post-mortem infiltration, can be mistaken for this appearance.

We attribute a great deal of importance to this suggestion, and consider it the most practical and satisfactory of any we have seen.—*Medical and Surgical Reporter.*

Worm Mixture.—

| | | |
|---|---|---|
| R.—Worm Seed Oil, | . . . | f oz. j.—M. |
| Turpentine Oil, | . . . . | f oz. ij. |
| Castor Oil, | . . . . | f oz. iij. |
| Lime Water, | . . . . | f oz. x.—M. |

Shake well.

Dose for a child one year old, one teaspoonful; for an adult, two teaspoonsful. Give three days in succession, and follow with cathartic, if required.

Resorts of Consumptives.—Dr. Lombard, in an Italian medical journal of recent date, *La nuova Liguria Medico*. June, 1872, has published an able essay, recommending for consumptives, and those threatened with weak lungs, a residence in cool and elevated localities, at least fifteen hundred feet above the level of the sea. Only such places should be chosen, however, as have comfortable accommodations, a generally clear sky, readiness of access, pleasant scenery, and the conveniences requisite for an invalid. Cold bathing, gymnastics, and open air exercise, abundance of milk, butter, nourishing fatty diet, good wine, and cod-liver oil, are the indispensable accessories. With these, the chances of a complete cure are very good, in the early stages.—*Med. and Sur. Rep.*

INJECTIONS OF ERGOT IN MALADIES OF THE UTERUS.—[Gazetta Med. Ital. Lombard., 1872, No. 4.]—Dr. Swideski, in a recent memoir, collects about forty cases in which subcutaneous injections of ergot were employed in various diseases of the womb, especially chronic metritis, in some displacements and in metrorrhagia; its action was in every case prompt and certain. The author used at first the solution of Beaujeau, and observes that, as the solution contains a larger quantity of alcohol, it acts more promptly, but excites greater pain, and frequently produces ulcers. The solutions employed are:

| | | | |
|---|---|---|---|
| 1. | Ext. aqua. secal. cornut., | . . | gram. 2. |
| | Spir. vin. rectif.,. | | |
| | Glycerina, | . . " aa | " 7 |
| 2. | Extr. aqu. secal. cor., | . . | gram. 2 |
| | Spt. vin. rectif., | . . . | " 5 |
| | Glycerina, | . . . . | " 12 |
| 3. | Extr. aqu. secal. cor., | | |
| | Spt. vin. rectiff., | . . . | gram. 2 |
| | Glycerina, | . . . . | " 12 |
| 4. | Extr. aqu. secal. cor., | . . | gram. 1 |
| | Spt. vin. rectif , | . . . | " 1 |
| | Glycerina, | . . . . | " 3 |
| | Aq. destil., | . . . . | " 4 |

In cases of chronic ulceration he employs solutions 3 and 4; where prompt action is needed, Nos. 1 and 2; the former (Nos. 3 and 4) act in from one to two hours, the latter excite painful contractions in about half an hour. In some of the deviations of the womb the author reports the favorable results of the use of subcutaneous injectious of ergot; he observes, however, that it is of no benefit if the case is of too long standing, if the uterus presents considerable inflammation, or adheres to other organs in the vicinity. In chronic metritis the injections are employed every two or three days, and, even where a complete cure cannot be hoped for, the leuchorrhœa speedily ceases, and the uterus returns to its place.—*New York Medical Journal.*

OZOKERIT IN SKIN DISEASES.—H. S. Purdon, M.D. (Dublin Quarterly Journal of Medical Science), recommends ozokerit, or vegetable wax, in the treatment of chronic affections of the skin, eczema of long standing, unaccompanied by much infiltration of the subcutaneous cellular tissue, tinea, scabies, and psoriasis. He states that it is as valuable as carbolic acid, oil of cade, or tar. Its action is regarded as that of a stimulant to the skin.—*Medical Record.*

SUPPURATION OF ONE-HALF LOBE OF CEREBRUM.—Dr. Schartzenthal relates briefly the case of a man 30 years of age, a day laborer, who had suffered for over two weeks, in the commencement of May, in the year 1871, from pain in the head, with languor and want of appetite, followed by a severe fever of an entire month's continuance. After this, apparent convalescene ensued, and the patient resumed his ordinary occupation. About four weeks he had left the hospital, whilst engaged in an altercation, he received a blow upon the head, and instantly expired. Examination after denth showed that the posterior half of the right lobe of the cerebrum was reduced to a circumscribed accumulation of pus, while the anterior half of the lobe and the entire left cerebral hemisphere were of a doughy consistence. The cerebellum was to some extent softened. Up to the period of his death, the patient had continued at labor without apparently the least difficulty.—*Centralblatt f. d. Med. Wissenschaften*, 1871, No. 12 from the *Wien Med. Presse*, 1871.—*Amer. Jour. Med. Sciences.*

BLEACHED TINCTURE OF IODINE.—The Pharmacist and Chemical Record (July, 1872) gives the following formula for the above preparation:

R.—Tinct. of iodine,
Glycerine, pure, . . . aa oz 1.
Sulphite of soda, . . . dr i.

Rub the salt to a powder in a small mortar, and add the glycerine gradually; then pour in the tincture of iodine and triturate gently until a solution is effected, and the mixture assumes an amber color.

It is claimed that the properties of iodine are increased by the addition of the sulphite of soda, and that the glycerine enhances its value and convenience for local application.—*Medical and Surical Journal.*

DIFFERENTIAL DIAGNOSIS OF ANÆMIC FROM ORGANIC MURMURS OF THE HEART.—Dr. Jas. H. Hutchinson (Philadelphia Medical Times, Aug. 15, 1872), in a lecture on anæmia, states that he has found a peculiarity in cardiac murmurs arising from anæmia, which is but obscurely alluded to by some writers on auscultation. The murmur will be found to be much more intense when the patient is in the recumbent position than when he is either standing or sitting. Having never failed to detect this greater intensity in the recumbent position in every instance in which he has auscultated anæmic patients, Dr. H. believes it is a characteristic of some importance in the differential diagnosis of anæmic from organic murmurs.—*Ibid.*

EFFECTS OF BAD AIR ILLUSTRATED.—A striking example of the effects of vitiated air is detailed by Dr. R. A. Smith, of London, in Nature. It sets in a clear light the fact that no great diminution of the percentage of oxygen in the air is necessary to make an atmosphere almost deadly to human beings, and the fact that we may be about as ignorant of the noxious quality of the air we are breathing, even while we are taking it into our lungs, as we are susceptible of injury from it. In some of his experiments he used an air-tight chamber, the atmosphere of which he vitiated by burning candles in it. On one occasion a young lady, who was anxious to make the experiment, got into this enclosure just as the candles were put out, and at the end of five minutes, during which she made light of the difficulty, it was necessary to help her out, the partial asphyxiation having taken place in an atmosphere in which the percentage of oxygen was about 19.0.—*Medical and Surgical Reporter.*

PILL FOR NEURALGIA.—

R.—Ext. Hyosciamus,
Ext. Valerian,
White Oxide of Zinc, . . aa dr. j.—M.

Make the mixture into sixty pills. One pill may be taken every three hours. This is a favorite old prescription of the German and French physicians, and as mentioned in many of their works on therapeutics.

SULPHITE OF SODA IN TENIA CAPITIS, ETC.—In the American Journal of Medical Science for October, 1869, Dr. Charles M. Watrous, of Pennsylvania, recommends the sulphite of soda, as a local application in tinea capitis, crusta lactea and similar affections. In one case he used half an ounce to a pint of water, applied constantly by compresses wet with the solution—making it weaker, as it caused smarting. In another case he used forty grains of the sulphite to half an ounce each of distilled water and glycerine, the parts moistened with this three or four times a day. This last prescription he has used in the ear three times a day in scrofulous otitis, dropping in a few drops after washing the ear out, and excluding the air by cotton wool.

ANODYNE LINIMENT IN OTITIS.—

R.—Ext. Belladonna, . . . gr. x.

Aqua Dist, . . . . . . oz. ss.

Glycerine, . . . . oz. ss.—M.

S. A cotton ball soaked in the mixture to be placed in the external auditory canal. M. T.

*Neuralgia of the Testis.*—In the majority of cases, says Dr. Lazarus, in the Wiener Medical Presse, July, 1872, neuralgia of the testis is caused by abstinence from coition. Not in real celibates, however, but in those accustomed to indulge their passions, and who for any cause are prevented from doing so for a long time. It is often relieved by an involuntary nocturnal emission, or by a half successful coitus. (?) Unsatisfied sexual excitement produces it; hence its vulgar name, "horn colic."

As a very efficacious remedy, the Doctor recommends sulphate of zinc, both internally and hypodermically, in the usual doses. For the stomach, it may be combined with a little hydrocyanic acid.—*Medical and Surgical Reporter.*

*Turpentine Mixture.*—Dr. E. Hurd (Eclectic Medical Journal, August, 1872) says: For the following mode of giving the oil of turpentine, I am indebted to my friend, Dr. S. H. Potter, of Hamilton, Ohio. I have found it of great service in gastro-intestinal irritation:

| | |
|---|---|
| R.—Powdered Gum Arabic, - - - | dr. jss. |
| Peppermint Water, - - - - | oz. j. |
| Castor Oil, - - - - - | oz. j. |
| Spirits Turpentine, - - - - | dr. jss. |
| Ol Anise, - - - - - | gtts. v. |
| Syrup Rhei, - - - - - | oz. iss. |
| Syrup Ipecac, - - - - | oz. ss.—M. |

Make an emulsion. A teaspoonful every hour to children; a tablespoonful to adults.

*Bromo-Chloralum.*—Dr. Cowling reported (Proceedings of College of Physicians and Surgeons, American Practitioner) that he had used this new agent as a disinfectant in the following cases among others, with the most satisfactory results: an enormous cancer of the face and upper jaw; abscess of the thigh after amputation; a large and very foul acscess between the shoulders.

*Adulteration of Aniline Colors.*—According to Joly, sugar is now extensively used to adulterate the aniline colors. It can best be detected by treating the dye with a mixture of alcohol and ether, which dissolves the aniline and leaves behind the sugar.—*New Remedies.*

*Bromine as an Oxidizing Agent.*—According to P. Waage, bromine is a very energetic oxidizer of all other substances, and will convert the sulphuret of a metal into sulphuric acid and the oxide of the metal.—*New Remedies.*

On the Employment of Creasote in Deafness,—By Dr. Harrison Curtis.—One of the principal causes of deafness is the absence of the secretion of cerumen in consequence of a fault in the action of the ceruminous glands. Often in my clinics, even when the deafness has continued for a long time, I have observed that it has no other cause, and on removing that I have caused the infirmity to disappear. It is very true that to obtain this result, more or less time is necessary, according to the duration of the infirmity, and in proportion to the gravity of the first cause of the inaction of the glands. After having cleansed the auditory meatus, and re-opened, so to speak, the orifice of the passage, by removing the morbid secretion which obstructs it, the use of a moderate stimulant is indispensable to re-establish the normal action of the glands. But before all, it is necessary to cleanse the auditory meatus, as no remedy can have the least effect, unless this operation has been well performed. In general I employ a preparation composed of half an ounce of beef's gall and a drachm (*un gros*) of tincture of castor or tincture of musk. With it I moisten a piece of cotton, which I place in the auditory meatus at night, to soften the hardened cerumen. In the morning I syringe the ear with warm water, to which may be added an ounce of soap liniment and a little cologne. I have often substituted with advantage, for the preparation of beef's gall and tincture of castor, the solution of potass of our pharmacopœa (London ?) with the oil of sweet almonds, to dissolve the cerumen.

I would recommend for this operation, to be particular in the choice of a syringe. When the ear is well cleansed, and the glands are in such a state that a stimulant can act upon them, I would advise, in accordance with results which I have obtained from my clinical experience, the employment of a solution of creasote in oil of (sweet) almonds, to induce the ceruminous glands to resume their normal action. The following is the formula which I employ:

R.—Creasote, - - - - - f. dr. j.
Oil of Sweet Almonds, - - - f. dr. iv.—M.

And with a badger's hair-pencil put a small quantity in the auditory passages night and morning. I ordinarily commence with a solution of this strength, and augment the quantity of creasote according the effects obtained. Cases, however, present themselves, in which no good result will be obtained from this application without applying behind the ear a vesicatory ointment of tartarized antimony, or other derivatives. In otorrhœa, and always when there is pain or inflammation the creasote is contra-indicated.

Its application causes no pain or unpleasant sensation, but only an agreeable feeling of warmth.—*Lancet.*

THE above article was published many years ago, but as the Senior Editor has used the treatment recommended with great success in many cases, we re-publish it for the benefit of those who have never read it.

WILL SULPHUR PREVENT CONCEPTION?—Dr. H. J. Smith, of Blackshear, Pierce county, Georgia, narrates two cases, occurring under his own observation, where sulphur administered internally seemed to prevent conception. In the first case the medicine was given for hemorrhoids, and the patient, though cured of this disease, persisted in its use. Quite prolific up to the time she commenced the sulphur, she ceased to bear children; and this immunity from pregnancy has continued for six years. The second case is essentially similar, except that the sulphur was taken solely for the purpose of preventing conception.

If the devil—the printer's devil, of course, is meant—were at our elbow, he would, doubtless, suggest a future administration of sulphur to all who strive to violate the laws of maternity.—*American Practitioner.*

INGROWING TOE-NAIL.—Dr. Finch writes to the British Medical Journal:

Neither of the cutting operations is at all necessary for the complete and rapid cure of ingrowing toe nail. If a small, thin, flat piece of silver plate be bent at one edge into a slight deep groove, and after the toe has been poulticed twenty-four hours, slipped beneath the edged of the nail, so as to protect the flesh from its pressure, and the rest of the thin plate bent round the side and front of the toe, being kept in position with a small portion of rosin-plaster passed round the toe, a speedy and almost painless cure will take place; and the patient, after the first day, has the additional advantage of being able to walk. I have followed this method in numerous cases with uniform success.—*Medical and Surgical Reporter.*

ARBOR VITÆ IN SMALL-POX.—The leaves of Thuja Orientalis and Occidentalis are employed in Belgium against small-pox. A tincture is prepared by macerating, for 10 days, 1 part of fresh leaves with 10 parts of 90 per cent. alcohol; it is given in water in doses of 10 drops.—*Eclectic Medical Journal.*

SOOTHING SYRUPS.—Dr. Scudder (Eclectic Medical Journal) asks: What shall we use in the painful ailments of childhood? Where will we find a "soothing syrup?" I will answer these questions briefly:

Using medicine in the olden way, I have prescribed with excellent results:

| | | |
|---|---|---|
| R.—Comp. Spirits Lavender, | - - - | dr. iij. |
| Tinct. Lobelia, | - - - - - | dr. j. |
| Simple Syrup, | - - - - - | oz. j ss. |

Dose, from ten drops to a teaspoonful. There is nothing harmful in this prescription, and it will be found an excellent "domestic medicine." It is "good for colic, for colds, slight fevers, etc., and will give satisfaction.

You have doubtless heard of "catnip," possibly of "old catnip," which we don't want, for we always prefer our medicines from the *fresh* herb. Take of catnip leaves and tops when in full bloom and possessing the strongest odor, a sufficient quantity; thoroughly bruise with your wife's "potato masher," and pack in a percolator, cover with proof spirit; let it stand for four days, then draw off, adding spirit until you have a pint of percolate for each eight ounces of herb. This tincture in the proportion of one to three of simple syrup, will be found an admirable "soothing syrup," and one of the best diaphoretics in the Materia Medica.

In ordinary practice I find my "soothing syrups" in aconite and nux vomica. If a child is feverish and has pain or is restless I give aconite. If there is an irritable state of the bowels, with greenish stools, ipecac is added.

But for *colic* nux is the remedy, and I rarely prescribe anything else. One drop to the ounce is the proportion for childhood, the dose according to age, from a few drops to a teaspoonful. It is one of those things that "work like a charm," and satisfies both patron and physician.

THE LATEST CURE FOR CHOLERA.—We notice in the Allgemeine Medicinische Central-Zeitung, August 14, the latest cure for cholera. It appears that the President of the Belgian Academy of Medicine has received a communication from Taurus, relating that the Persian physicians have been using with signal success, in the present cholera epidemic, a drink called Ghiacourt, made of milk curdled with rennet. This is mixed with water and ice, and given in small quantities repeatedly.

The juice of unripe grapes with ice has also, it is said, been used with good results, though this latter, to our mind, would be more likely to produce than to cure the cholera.

A SIMPLE METHOD OF ARRESTING EPISTAXIS.—While resident at the Philadelphia Hospital, I resorted to the following plan of arresting epistaxis, with entire success:

I was called into the medical ward one night to a patient bleeding profusely from the nose, the simple measures usually resorted to—as cold, solution of tannic acid, alum, etc.—having failed to control it. Not having any of the more efficient means usually employed for the arrest of such a hemorrhage, and seeing the dry tannic acid on the table, I remembered the directions given in cases of infantile coryza by Dr. Albert H. Smith, of this city, for softening the hardened secretion in the nostrils. He recommends the introduction of lard upon a roll of fine linen wrapped like an ordinary lamplighter.

It occurred to me that a similar roll of paper, moistened with water and coated with the dry tannic acid, inserted into the nose might be of service. I tried it, with immediate success.

I have since found that old linen answers the purpose better than paper applied as above, as it makes a better carrier, being softer, more flexible, and less liable to break down through excess of moisture. I have also found that the powder adheres better if soft lard be used instead of water.

Any powdered styptic may be employed in the same manner. This plan presents the advantages of being always practicable, and of bringing the powder directly in contact with the mucous membrane without danger of wounding it or of breaking down the delicate turbinated bones.

I have tried this repeatedly with uniform success, and believe, if it were resorted to, that the disagreeable operation of plugging would seldom be found necessary.—*Roland G. Curtin, M. D.*

FOR LUMBRICOIDES—

R.—Santonine, - - - - - - gr. ij–v.
Pulv. Jalap, - - - - - grs. v.—M.

To be taken at one dose at bed-time.

R.—Fluid Extract Male Fern, - - - dr. iss.

To be taken fasting; follow in one hour with two ounces Oil of Turpentine in emulsion with egg. For Tape-worm, it is a certain remedy.

AMMONIA FOR SNAKE-BITES.—Australian papers contain accounts of several cases of recovery from the bite of poisonous snakes due to the adoption of Prof. Halford's plan of the injection of ammonia into the veins. This succeeded in several cases after other plans of treatment had been tried without any good effect.

Alcoholic Treatment of Wounds and Atonic Sores.—Dr. Thomas Cooke recommends the following method:

1. The abscess is opened in the usual way; any clean knife will do for the purpose.
2. The wound is to be washed first with tepid water, then with the spirit-lotion.
3. A pad of lint or some tow dipped in the lotion is to be applied to the wound, and, if the cavity of the abscess be large, partly introduced into the same.
4. A piece of gutta-percha tissue or oil-cloth is to be placed over the lint or tow, and a common bandage is to be applied. The "spirit-lotion" used by Dr. Nelaton is "alcohol camphre," which is nearly similar to the spiritus camphoræ of the British Pharmacopœia; Dr. See uses pure undiluted alcohol; Dr. Dolbeau, the same diluted with half water. My spirit-lotion is composed of equal parts of methylated spirits and common water.

The carbolic system is, as a rule, never applied in any thing like the careful manner prescribed by Prof. Lister. Were it so applied, or rather, were it possible that it should be, no dressing, I believe, could supersede it. But where is the busy general practitioner, where is the surgeon to the out-patient department of almost any public charity, who could afford time to attend one-half of the minute details above mentioned! Now, Prof. Lister's plan has utterly failed wherever any one of these has been neglected. Hence its almost complete abandonment.

The spirit-dressing is easy of application; the lotion I use is inexpensive; its therapeutical effects are very similar and almost equal to the best results ever obtained by the carbolic system. May I beg to recommend my dressing?

The Castor-Oil Emulsion.—

| | | |
|---|---|---|
| R.— | Castor Oil, - - - - - | oz. j. |
| | Sweet Milk, - - - - - | 1 gill. |
| | Ess. Cinnamon and Sugar, - - - | q. s.—M. |

Boil a few minutes. To be taken at one draught. The taste of the castor oil is completely disguised.

Syphilis—Iodides of Ammonia and Sodium.—It sometimes happens that, in cases of later secondaries, or tertiary syphilis, requiring the prolonged use of iodide of potassium, the drug ceases to produce any result, or is no longer tolerated. This may be possibly owing to the deteriorating action of the base on the blood. Ammonium and sodium are not open to the same objection, and iodides of either of these bases should be substituted.—*Braithwaite's Retrospect.*

Cider and Rheumatism.—It has come to light that in certain parts of England, working people and even little boys employed to frighten birds away are compelled to take a considerable part of their wages in cider. Common laborers are requirad to take three pints per day, carters and shepherds four pints, and boys one pint. Women who work out are required to take one quart daily. Aside from the moral evils of the practice, it is found that the health of the laborers is seriously affected, and that rheumatism is a frequent result; as might be expected on the prevalent theory that that disease is caused by, or is associated with, lactic or uric acid in the blood. The subject has enlisted the attention of the House of Commons, and proceedings have been instituted for the purpose of correcting the evil.—*Pacific Medical Journal.*

Blisters in Orchitis, Chronic Gonorrhea, etc.—D. W. H. Galt (Proceedings of College of Physicians and Surgeons), recently saw a case of swelled testicle which, after resisting the usual remedies, had yielded very quickly to a blister applied on the inside of each thigh, as recommended by Dr. Furneaux Jordan. He had found blisters applied in the same locality exceedingly efficient in chronic gonorrhea. A case of threatened abscess of the labia had been averted by blisters used as stated.

Gonorrhea—Carbolic Acid.—The following is an excellent injection in gonorrhea: Carbolic acid, eight grains; tannic acid, eight grains; glycerine, half an ounce; water to one ounce.—*Braithwaite's Retrospect.*

Healing of Ulcers by Serum.—I have had several opportunities, during the year 1871, of trying the method I explained the previous year, of healing ulcers by covering them with serum. I propose to enlarge the observations during the coming year, and vary the methods already employed. Several striking results have, however been got. In one case, for example, a sore the size of a penny was healed in forty-eight hours; in another, one of three ulcers, each about the size of a florin, was experimented upon, and closed in three days, while the other two, in all respects similar, but treated by "water-dressing," remained unchanged. In another case, four hours and a half sufficed to produce a thin bluish covering of epithelium, like the "healing line" along the edge of contracting sores. Considerable care is requisite to ensure success, as the fluid must be carefully protected from contact till it "sets." When these experiments are complete, I will give an account of them.—*Glasgow Medical Journal—Canada Lancet.*

Plugging the Rectum for Hemorrhage.—William Allingham, F. R. C. S., Surgeon to St. Mark's Hospital for Fistula, London, (in his work on Fistula, Hemorrhoids, etc.) recommends the following procedure if secondary bleeding occurs after an operation for piles. When called to a case of hemorrhage, always arm yourself with a full-sized, bell-shaped sponge, and plenty of cotton wadding. Take also some persulphate of iron or powdered alum. Thread a strong silk ligature through near the apex of your cone-shaped sponge, and bring it back again, so that the apex of the sponge is held in a loop of the thread. Then wet the sponge, squeeze it dry, and powder it well, filling up the lacunæ with the iron or alum. Pass the forefinger of the left hand into the bowel, and, upon that as a guide, push up the sponge, apex first, by means of a metal rod, bougie, penholder, or a rounded piece of wood, if nothing better can be had. The sponge should be carried up the bowel at least five inches, the double thread hanging outside the anus. When this is so placed, fill up the whole of the rectum below the sponge thoroughly and carefully with cotton wool well powdered with the alum or iron. When the bowel is completely stuffed, take hold of the silk ligature attached to the sponge, and while with one hand you pull *down* the sponge, with the other hand push *up* the wool. The joint action will spread out the sponge, like opening an umbrella, and bring the wool compactly together. The plug should remain in at least a week, and it may be retained a fortnight or more.—*Medical Record.*

Nitric Acid in Pertussis.—

| | |
|---|---|
| R.—Acid Nit. dil., . . . . . | f dr xij |
| Tinct. Card. comp., . . . . . | f dr iij. |
| Syrup simple, . . . . . . | f oz iijss. |
| Water, . . . . . . | f oz j.—M. |

Of this, a teaspoonful is given as a dose every hour or every second hour, to be followed with a gargle of solution of carbonate of soda to prevent the action of the acid upon the teeth.

A Useful Form of Disinfectant has been recently brought into use in England, namely, sawdust soaked in a saturated solution of carbolic acid. It is convenient for many purposes, cheap, easily prepared, and not liable to be swallowed accidentally, as ordinary liquid disinfectants are.

Relation of Syphilis to Insanity.—Dr. Mildnen, director of the Lunatic Asylum in Klosternenberg, Austria, states that of 5,529 patients received at that institution during the period of fourteen years, in only seven cases, or 1.2 per cent., could the occurrence of insanity be traced as due to syphilis.

VALUE OF MYRRH.—Dr. de Savignac, in speaking of this drug, remarks, that he has seen painful dyspepsia rapidly relieved, and even cured, by the use of this gum-resin, either prescribed alone or in combination with other medicines, as bismuth, bicarbonate of soda, etc. Myrrh in these cases relieves the pain, awakens appetite, gives tone to the stomach, and renders digestion more active and regular. Besides these actions, it proves a general tonic in those gastralgiæ that are connected with various chronic diseases, and particularly with anæmia and chlorosis. But this last is often accompanied by amenorrhœa, and as strength returns the catamenia reappear. Cullen regarded myrrh as being only indirectly an emmenagogue; but Sydenham and many others have thought it exerted a direct action on the uterus. Its pectoral properties, he thinks, have been exaggerated, though it may aid other remedies in calming the cough and facilitating expectoration. He has found it particularly useful in vaginal and uterine catarrhs, and especially when applied topically in the form of vaginal injections, containing one to three drachms of tincture or vinegar of myrrh in a quart of water. Mattiolli and some others have prescribed it with success in intermittent fevers, but further evidence is required on this point. Alibert admits its value as a topical remedy in skin affections, and it has been largely used for ulcers, for the dressing of wounds, and cases of caries and necrosis, and as an antiseptic in wounds of the soft parts of a bad nature, likely to become gangrenous. For this purpose the tincture of myrrh is best adapted. As an internal remedy, acting as an excellent stomachic, he strongly recommends the following prescription for the wine of myrrh:

| | |
|---|---|
| Picked myrrh, . . | 20 grammes. |
| Rind of Seville oranges, . . | 15 " |
| Malaga wine, . . . | 1½ pint. |

Macerate for ten days and filter.

THE TINCTURE OF EUCALYPTUS AS A DRESSING FOR WOUNDS.—M. Demarquay, in the course of some experiments at the Maison Municipale de la Sante of Paris with the tincture of the leaves of this plant, which has a very agreeable odor, and is believed to possess disinfecting, antiseptic and healing properties, found that it often gave good results in cases in which the more usual remedies had failed. He adds that the tincture acted not merely by disinfecting and ameliorating the surface to which it was applied, but that its effect in removing the fœtid odors which had rendered the patients unable to eat or sleep was still more beneficial.—*Philadelphia Medical Journal.*

On the Use of Ergot of Rye in Dysentery.—M. Luton, of Rheims, states (Gaz. Hebdomadaire, Oct. 1871) that during an epidemic of dysentery which lately prevailed at Rheims, he employed in the cure of the disease most of the remedies which are considered efficacious, and met with various degrees of success. The epidemic was not a very severe one, and most of the patients recovered; but, in the majority of the cases, it appeared to M. Luton that the therapeutical action was not very evident, nor the relief rapid; and moreover some of the patients, especially among the most aged, died. It was therefore desirable that some new method of treatment should be found, which might give more satisfactory results, and the opportunity of doing so was offered by the case of a female patient, who was suffering at the same time from uterine hemorrhage and dysentery. The ergot of rye was prescribed successfully for the former malady, and it was found that the latter was likewise benefitted by the remedy; and, in fact, as soon as the first doses had been given, a condition of constipation was induced, which lasted for four or five days. This first experiment led to the use of the ergot in simple dysentery, and it was found that an improvement in the symptoms, and eventually a complete cure, followed the new plan of treatment. M. Luton gave the ergot in powder, in the dose of 3 grammes (about 45 grains) a day, divided into doses of 50 centigrammes (abous 7½ grains); and two or three days generally sufficed for a cure in ordinery cases. The ergot appeared to attack not only the hemorrhagic element of dysentery, but the whole disease; and the mucous secretions, the griping, colic, and fever, were equally relieved at the very commencement of the treatment.—*Brit. and For. Med. Chir. Rev.*, April, 1872.

A Good Nitro-Muriatic Bath.—

R.—Nitric Acid,
Muriatic Acid, . . . . aa oz i.

Mixed together in a pint of water. Put enough of this mixture into the quantity of water designed for a bath to make it as sour as strong vinegar. Use hot or cold water, as the patient may prefer. It should be strong enough to make the skin itch or tingle. W. H.

Sulphite of Soda in Variola.—Dr. Maurice Pflaum, of Pittsburg, Pa., comes to the conclusion that sulphite of soda, given at any time before the eruption has fully come out, will invariably cut short the disease, even more effectually than quinine does intermittent fever.

Dogwood.—In reference to the case of poisoning in Missouri from the use of Peruvian bark and dogwood bitters, a correspondent suggests that physicians should be careful in prescribing and druggists in putting up powders and extracts of dogwood. The American dogwood is astringent, aromatic and tonic, and is valuable as a remedy in low and intermittent fevers. The introduction of quinine has, however, nearly banished this substitute for cinchona from practice, except as a domestic remedy. There is also a dogwood, a native of the West Indies, bearing the commercial name of "Jamaica Dogwood," which is a powerful narcotic, and is used in poisoning fish. The accidental substitution of the latter of these remedies for the American dogwood would be attended with serious and, in many cases, fatal results.

Animalcules in Buttermilk.—Dr. J. P. Brown, of Galt, Ontario, in the Canada Lancet for August, gives an account of a family poisoned by drinking buttermilk, which, on examination with the microscope, was proved to contain immense numbers of animalcules. He thinks the germs which produced them were taken in by the cow in the water she drank, and found their way into her blood through the lacteals. The symptoms were those of narcotico-irritant poisoning, viz: vomiting, purging, cramps, stupor, etc. We think it much more probable that the organisms were produced in the milk after it was taken from the cow.—*Pacific Medical Journal.*

Chloral-Hydrate in Spasmus Glottidis.—[Jahrbuch fur Kinderkrankheiten, 4. H., 1871.]—Dr. Rhen treated a case of spasmus glottidis, which had assumed a threatening character through the severity and frequency of the attacks, with chloral-hydrate. The attacks were perceptibly lessened and finally quieted. The child thus treated was seven months old. The medicine was well borne, and produced no disturbances of digestion.

Oxide of Silver in Menorrhagia.—

R.—Argent. Oxid., . . . . grs. jss.
Ext. Tarax.,
Tragacanth pulv., , . . . aa grs. v.
M. f. Pill. Na. 6. One to be taken each day.

Occupation in the Causation of Skin Disease.—Mr. Balmanno Squire, of London, an acknowledged authority on skin disease, affirms that the majority of the cases are found among book-binders, dyers, tanners, hatters, weavers, leather cutters and fur-workers, all of whom have much to do with animal products.

THE WARM BATH IN SMALL-POX.—Dr. Stokes, Regius Professor of Physic in the University of Dublin, recommends, in the Dublin Journal of Medical Sciences, for January, 1872, the use of the warm bath in the treatment of small-pox. He says: "We cannot doubt that the mortality in small-pox hospitals would be greatly diminished by the use of the bath." After describing a very severe case of confluent small-pox, in which the patient is kept alive only by stimulants, he said the trial of the warm bath was suggested to him by Mr. Smyly. "The effect was instantaneous and marvellous. The delirium ceased as if by magic. It was the delirium of pain; and the patient exclaimed: 'Thank God! thank God! I am in heaven! I am in heaven! Why didn't you do this before?' The fetor immediately and completely disappeared, so that, on entering the ward, no one could suppose that there was a case of small-pox in it. He was kept at least seven hours in the bath." This case, and its singular result, in addition to the experience of Hebra, justify the recommendation of the use of the bath.—*Philadelphia Med. Times.*

ANTI DYSPEPTIC PILL IN CASES OF ATONIC DYSPEPSIA.—

| | |
|---|---|
| R.—Potass. sulph. | oz ij. |
| Cubeb pulv. | oz j. |
| Ginger pulv. | |
| Rhei. pulv. | aa oz ss. |
| Oil Gaultheria, | gtt xx. |
| Mucilag. Accaciæ, | q s. |

M. H. Pill. No. 70.

Dose one before each meal."

It will doubtless be of value in many cases which require a mildly stimulating laxative to aid a feeble digestion.—*New Hampshire Jour. of Medicine.*

RAILWAY AIR-CUSHION.—Persons unused to travel often suffer much fatigue in the lower limbs from a long journey by rail, which is attributed to the tremulous motion communicated from the floor of the car. Those who have experienced the difficulty there is in walking over a bridge or a loose plank road while a vehicle is passing by, and the painful effort required to keep the legs in trim, will appreciate the case. As a preventive, it is recommended to rest the feet on an air-cushion. Footstools of this kind are made for the purpose. All invalids and persons unused to hardships would do well to supply themselves with this simple contrivance before entering on the overland journey by rail.—*Pacific Medical Journal.*

NICOTINE IN TETANUS.—Dr. E. M. Wath relates, in the Australian Medical Journal for September, 1871, a case of acute tetanus, marked by "terrible opisthotonos," in which he injected into the areolar tissue of the thigh half a drachm of a solution, one drop of pure nicotine in an ounce of water. The pulse immediately sank to sixty; all the muscles relaxed, except those of the neck; and presently the patient began to perspire. He fell into a sound sleep for four hours, after which he had fully recovered speech. On awaking he said that he felt "all benumbed." He then took some wine and water, and an egg beaten up in milk. The injection was used at twelve o'clock at night. The patient awoke about four o'clock next morning. One hour later a pill of one grain of extract of nicotine of the Hessian Pharmacopœia was administered to him. He again awoke drowsy at nine A. M. Although his body felt very sore, the tetanic symptoms had ceased, and from this time his bowels and bladder acted regularly. A pill of the same extract was given for three nights following, after which the patient felt and continued well.—*Medical and Surgical Reporter.*

A GOOD PRESCRIPTION IN FACIAL NEURALGIA.—

| | |
|---|---|
| R.—Distilled water, . . . | f oz ij. |
| Valerianic acid, . . . . | f oz j. |
| Subcarbonate of ammonia, . . | q. s., |

to neutralize the acid, add,

| | |
|---|---|
| Alcoholic extract of valerian, . . | scr ij. |

Dose, one teaspoonful three times a day.

VEGETABLE GROWTHS IN THE EAR.—Since the year 1844, when the attention of the profession was first called to the subject, the growth of minute fungi in the ear has been reported to be a common cause of disease of that part. The meatus, canals, and tympanum are sometimes covered with the growth in the form of white or yellow mold on their surfaces. Prof. Seely of Cincinnati reports in the "Clinic" three cases of diseased ear in which he detected the fungus Aspergillus. Tinnitus, inflammation, and the accumulation of wax are the attendant symptoms. The treatment consists of the application of a solution of carbolic acid, five grains to the ounce of water. As it is found impossible to transplant the ectophytes to a healthy ear by inoculation, we suspect they are the effects of disease rather than the cause.—*Pacific Medical Journal.*

Sleeping to Death.—The British Medical Journal (Aug. 10, 1872,) refers to the singular and always fatal and lethargic disease (*lethargus*) occasionally observed to be prevalent among the negroes of West Africa.

Dr. T. H. Bailey, who has observed it there, but who is not able, more than others have been, to explain its pathology, describes it briefly, as follows: It is one of the curiosities of medicine. As the name implies, the principal, and, in fact, only symptom that presents itself is letharthy, and one case is essentially a stereotype of all. The patient, usually a male adult, is seized without any premonitory symptom, with a sensation of drowsiness, which continues rapidly to increase, in spite of all efforts to throw it off, until he sinks into a profound and seemingly natural sleep. This continues for about twenty-one days, when death takes place. Throughout the course of the disease, the patient preserves a quiet and peaceful countenance, may be easily aroused for a short time, will take nourishment, and generally answers a few questions in a perfectly rational manner. The pulse, respiration and temperature, remain normal throughout; the pupil is neither dilated nor contracted to any noticeable extent, and the urine and fæces are voided with comparative regularity. With the exception of the abnormal tendency to sleep, nothing exists to denote disease." Many careful *post-mortem* examinations have been made by competent men, but nothing of an abnormal character has been found. Dr. Smith, colonial surgeon at Freetown, says that every remedy that could possibly be of any avail had been used, without any apparent beneficial effect. They sleep on and glide into eternity, in spite of professional skill.—*Med. and Surg. Jour.*

Sore Nipples.—

| | |
|---|---|
| R.—Balsam of Peru, . . . | f oz j. |
| Sulph. ether, . . . | f oz iij.—M. |

This heals the fissures quickly and does not harm the child.

I give another formula which I think very good.

| | |
|---|---|
| R.—Pith of sweet almonds, . . . | oz ij. |
| Gum Arabic, . . . . | oz j. |
| Rose water, . . . . | f oz ij. |
| Balsam of Peru, . . . | f dr ss.—M. |

This is to be applied in the same manner as before directed.—N.

Chloride of Soda.—A mixture of one part with seven or eight of water has been successfully used in chilblains and burns, and also for bad wounds, especially for sores in which mortification has commenced.

THE PHOSPHATE OF SODA has two uses—as a restorative—and for its influence upon the intestinal tract. As a restorative I employ it extensively with children, in those cases where there is impaired nutrition, with pallidity of tongue and mucous membranes. In these cases it will be found to exert a markedly beneficial influence. It is generally administered in milk in doses of one to three grains, four times a day. We occasionally find a case in the adult where it will prove beneficial. These are uniformly marked by the pallid mucous membranes, and inaction of the bowels. The dose will vary from five to fifteen grains.

Its second use is as a laxative for children. We find cases of constipation that will yield to no remedies, the child suffers from indigestion, and occasionally from colic. In these cases phosphate of soda in doses of from three to five grains, three times a day, will give permanent relief.

Phosphate of soda is also an excellent laxative for the adult, especially in cases of habitual constipation, with hardened feces. In these cases twenty to thirty grains in a large glass of water is taken on going to bed at night.

OINTMENT FOR ITCHING OF THE ANUS.—

| | |
|---|---|
| R.—Glycerine, . . . . | oz 1. |
| Creosote, . . . . | gtt xx. |
| Starch, . . . . . | dr ii. |
| Oxide of zinc, . . . . | dr i.—M. |

S. Apply to the parts. M. G.

MAGNETIC WELLS.—Among the humbugs of the day are magnetic wells. They are found in various parts of the country, and are increasing rapidly in number. The magnetic quality of the water is ascertained by connecting a quantity of it in a vessel with the earth by means of a soft iron wire. The wire immediately gives signs of the presence of magnetism when tested by the magnetoscope. Such wells are proved to be highly medicinal, and a number of wonderful cures are already reported. Unfortunately, however, some one tried the magnetic experiment with other water, and found that the water of wells and springs in general displayed the same phenomenon. He went farther, and tried the empty cup, without any water, and still the magnetoscope told the same story. It is scarcely necessary to add that the effect is due to terrestrial magnetism, and that nearly all iron is more or less magnetic.—*Pacific Med. and Surg. Journal.*

Chloral in Asthmatic Bronchitis.—Dr. Caspar Morris says he was recently in attendance upon a lady who suffers from frequently recurring attacks of bronchitis, with asthma. The skin was hot, the frequency and difficulty of respiration very great, the rales loud and musical, and the secretion very profuse, so that the mucus could be poured from the cup in an abundant, ropy stream. His attention had been arrested by the account recently published, that the hydrate of chloral might be of service. He ordered five grains in one fluid drachm of the syrup of lactucarium of Aubergier, to be repeated in two hours, if required. The two doses afforded entire relief; and she has found great comfort since from a single dose taken at bed-time, a good night's rest being secured by it. He mentions it as a valuable aid in the treatment of this intractable and distressing disease.—*American Journal of Medical Science.*

For Cholera Infantum.—

| | | |
|---|---|---|
| R.—Bismuth subnit., | . . . . . . | dr. j. |
| Syr. zinzib., | . . . . . . | dr. iij. |
| Tr. cinnam., | . . . . . . | dr. j. |
| Tr. opii., | . . . . . . . | gtt. xviij. |
| Syr. acaciæ, | . . . . . . | oz. j.—M. |

Dose, a teaspoonful four times daily to children from one to two years old. To be continued until all disposition to vomiting disappears, and the fecal dejections diminish in frequency and become more consistent, which very generally obtained in from three days to a week, sometimes sooner.

Cerebro-Spinal Meningitis.—Dr. T. L. Wright (Cincinnati Lancet and Observer, July, 1872) says this seems to be a blood disease; specific, and *sui generis.*

If this is, indeed, a correct opinion, it follows, from what we know that the sulphites, and especially the bisulphite of soda, will prove prophylactic.

I am using this preparation in doses varying from one grain to ten or fifteen grains, twice a day, among my friends to prevent the disease from breaking out.

Laxative Pill—

| | | |
|---|---|---|
| R.—Podophyllin, | | |
| Leptandrin, | . . . . . | aa grs. ix. |
| Capsicum, | | |
| Sulphur, | . . . . . . | aa grs. viii. |

M. et fiat mass., in pillulas xii dividenda.

One pill may be taken every night, or often enough to keep the bowels soluble and regular.

Inflammation of the Kidneys Following the External Application of Tar.—Although the theory of metastasis in the treatment of skin diseases has been for a long time vigorously combatted by Prof. Hebra, there still lurks in the minds of certain writers of eminence, among whom may be mentioned Niemeyer, that where a morbid secretion of the skin is suddenly suppressed, some injurious effects may be produced upon the internal organs, owing, perhaps to the additional labor they are called upon to perform in eliminating this morbid matter.

In the case here reported by Dr. Kirchhelm, (*Beliner Klin Wochenschrift*, May 6, 1872,) very serious internal lesions were found to follow the cure of an eczematous eruption, but the unfavorable result is ascribed by that gentleman not to any metastasis of the eruption, but rather to the irritating effect upon the kidneys of the tar employed externally in the course of the treatment. This agent, when applied externally, is absorbed by the skin, and is eliminated by the kidneys, and Hebra is in the habit of warning students, that where an extensive portion of the body is smeared with any of the preparations of tar, pain in the kidneys and strangury may be produced. In no instance, however, does he appear to have met with any serious effects from this mode of treatment.

The patient of Dr. Kirchhelm was a robust young man who entered the hospital on account of an infiltrated eczema of the lower extremities, of about a month's duration. By the aid of the usual baths and applications of soap, oleum jecoris, unguentum diachylon, etc., the eruption was soon brought back to a condition of eczema squamosa, after which a preparation of oil of cade and glycerine was applied on three consecutive days. On the fourth day indications of general constitutional disturbance were noticed, such as headache, loss of appetite, pain in voiding urine, etc., so that the application of the tar was at once suspended. The symptoms instead of abating, however, increased in severity. Severe pain was now experienced in the region of the kidneys; the urine was reduced in quantity and loaded with albumen; the skin assumed an œdematous appearance, while the serous infiltration of the lungs was so great as to occasion great dyspnœa. After a dangerous illness, lasting nearly three months, and complicated by an attack of pneumonia, the patient had the good fortune to recover, completely relieved, by the way, of his eczema.—*Boston Medical and Surgical Journal.*

M. Barth, who, since Trousseau's death, has had a large share of the consulting practice of Paris, is President of the Academy of Medicine of Paris for 1872.

Fatal Case of Peritonitis Following a Single Examination of the Vagina.—Mr. Gillette reports [*Gaz. des Hopitaux*, *No.* 57, 1872; *Allg. Med. Cent. Zeitung*, July 6, 1872,] the following case of death from peritonitis, following a single vaginal examination, occurring in the case of a woman who had entered the hospital on account of a prolapse of the vagina. The patient was forty years of age, and desired admission to the hospital, complaining of general weakness and a feeling of weight in the perineum, attributed to a prolapse of the uterus. She had given birth to one child ten years previous. For about a year a profuse hæmorrhage had occurred at the menstrual epoch. After allowing her to rest for three days, a vaginal examination was made, at which it was ascertained that a moderate prolapse of the uterus existed. There was no indication that any pain was inflicted at this examination, nor was any attempt made to introduce a speculum.

On the following day a severe peritonitis ensued, the symptoms being all well marked, and the woman died in forty-eight minutes after the commencement of the attack. At the autopsy there was found, in addition to the usual changes of peritonitis, a decided congestion of the left Fallopian tube and *ostium abdominale.* The left ovary was congested and enlarged, while between it and the tube was found a cavity filled with pus, and lined with a false membrane. A small sub-peritoneal abscess was also found. These lesions, then, of the ovary and the neighboring parts formed the point of origin for the peritonitis, while the mucous membrane of the uterus was merely the seat of a fungus-like softening. It has been observed by Nelaton that there not unfrequently exists some latent affection of the appendages of the uterus, the result generally of chronic inflammation, or the presence of some fungous or fibrous growth, and that the abdominal pains induced thereby leads the patient to seek surgical advice. In these cases the simplest examination of the vagina may be followed by severe and even fatal results. Recamier, moreover, reports in this connection that two cases have come under his notice in which death ensued in forty-eight hours after some application had been made to the cavity of the womb.

These unfortunate instances should put the surgeon on his guard, when called upon to examine women, in cases where there may be any suspicion of latent disease in the appendages of the uterus, as indicated by the presence of pain in the iliac region. It should also be borne in mind that the instrumental examinations of the womb, when accompanied by pain, may be followed by the most serious results.—*Boston Med. and Surg. Jour.*

Cancer of the Tonsils.—Mr. Alfred Poland, in the course of a paper in the April number of the British and Foreign Medico-Chirurgical Review, after alluding to the great rarity of cancer of the tonsils, and to its inevitably fatal termination, says that it may be either primary or secondary, or of the medullary or scirrhous form. In regard to the diagnosis of the disease, he adds that "in the early stage of both forms of the disease there is no distinguishing mark to guide us as to the nature of the disease. Enlargement of the tonsil is the only sign, and this does not arrest the attention of the patient, nor excite any suspicion in the mind of the surgeon, in consequence of the very frequent occurrence of subacute and chronic inflammation of the glands in a very great majority of persons. As the disease advances, the peculiar nature of the fatal disease begins to develop itself. When rapid, it steadily encroaches upon the fauces and pharynx, involves the lymphatic gland at the angle of the jaw, and afterwards the cervical glands, and soon destroys the patient. The scirrhous variety, on the contrary, may often fail to be recognized; but its slow progress, and its becoming ulcerated and excavated on its surface, render it less liable to be confounded with chronic hypertrophy and syphilitic ulceration. However, both these diseases have passed for cancer; and, on the other hand, cancer has been presumed when subsequent results have disproved the supposition. Excessive hardness, implication of the lymphatic glands, peculiar ulceration, fetid discharges, increasing growth, and peculiar cachexia, seem to be its characteristics."

In speaking of the treatment, Mr. Poland says that, whenever there is any reason to believe that there is a syphilitic taint, iodide of potassium should be given, and in any case it might be well to give the patient the benefit of the doubt. He refers approvingly to Dr. Cheever's operation in extirpating an encephaloid tonsil and an enlarged gland at the angle of the jaw, by an external incision in the neck. This operation appears to have been performed, however, by Langenbeck in 1865, and by Huetur in the same year. Caustics, and escharotics generally, only aggravate the pain, and are not recommended. The removal by the amygdalotome is generally out of the question. The ecraseur may, however, be used, when the tumor has not attained a large size, and when the loop of the instrument can readily embrace the whole base of the tumor.—*Phil. Med. Times.*

Use of Sulphate of Quinine in Menorrhagia.—Dr. Deneffe reports (*Annales de Gand,* June, 1872) two cases from his own practice, confirming the opinion of Monteverdi and Bouque on the efficacy of sulphate of quinine as supplementary to ergot of rye.

The first was that of a young lady whose menses had usually been regular, but exposure to cold had suddenly suppressed them at the period immediately preceding her illness. Her period coming on again, the flow was abundant, and soon amounted to a menorrhagia. As she was away from home, a physician was called, who prescribed perchloride of iron, but without effect. Returning home to Dr. Deneffe, he ordered sulphate of quinine, to be taken in 1¼ gr. pills every hour; this almost immediately checked the hæmorrhage.

The second case was that of a lady whose menses had been suppressed for a while by the influence of violent emotion, and which afterwards re-appeared with a hæmorrhagic character, alarming the patient. She was confined to her bed, and the least movement increased the flow of blood. Sulphate of quinine was ordered, as in the previous case, and, the better to test the power of the drug, she was ordered to rise and walk about. The next day the menses had lost their hæmorrhagic character, and on the second day they had ceased entirely. These observations seem to support the theory of Monteverdi and Bouque that quinine acts as an excitant of the involuntary muscular fibres.

Ointment for Glandular Swellings.—

| | | |
|---|---|---|
| R.—Lard, | . . . . | 6 ounces. |
| Iodine, | . . . . | 1 drachm. |
| Calomel, | . . . . . | 25 grains. |

Triturate the calomel and iodine with a small quantity of the larn until well incorporated; then add the lard and mix. Effect, rubefacient and alterative.

Effect of Diet and Exercise on the Elimination of Nitrogen.—Parkes (Proceedings of the Royal Society, 1871, p. 349) finds that moderate exercise on a mixed diet has little influence on the excretion of nitrogen or on the temperature. It causes temporary quickening of the pulse, but this is succeeded by corresponding slowness, so that the mean pulse rate during the day remains the same. The elimination of nitrogen is increased during the period of rest after severe exercise. The muscles can obtain the energy required for great exertion from fat and starch, though certain changes in their nitrogenous constituents also occur, which give rise to increased elimination of nitrogen after the work is over.—*British Medical Journal.*

Transfusion.—Dr. W. S. Playfair reports (London Lancet, January 27) a case of transfusion in post-partum hæmorrhage, unsuccessful from return of hæmorrhage.

ARRESTING HEMORRHAGE AFTER EXTRACTING TEETH.—In the March number of this journal (vol. ix, p. 128), we published a method of arresting hemorrhage after the extraction of teeth, recommended by Dr. M. F. Shields. The American Journal of Dental Science copied the paragraph in the August number, and added:

"A better method, we conceive, and one we have been following for years past, is to saturate a pellet of cotton with sandarach varnish, and on the end of this take up a sufficient quantity of the powdered subsulphate of iron, which is carried direct to the mouths of the bleeding vessels in the alveolar cavity, immediately after this cavity has been cleansed of blood."—*Nashville Medical Journal.*

TREATMENT OF ASTHMA.—Asthma should, with a view to its successful treatment, be viewed as a neurosis of the pneumogastric nerve, of which, sometimes, the cause is disturbance of healthy function at the brain end, and sometimes at the gastric or hepatic. Thus bismuth and hydrocyanic acid are of great value when the neurosis is of gastric origin. Carlsbad salt, nitric acid, and at times, small doses of mercury, are all unmistakably curative when the hepatic system requires relief. Other remedies, such as ipecacuanha, belladonna, and nux vomica, are of use in appropriate forms of pneumogastric disturbance; whilst iodide of potassium, sulphur, and arsenic, are the remedies indicated if there is a gouty or rheumatic diathesis at the root of the malady.—*Braithwaite's Retrospect.*

PYROSIS.—Dr. Thompson (American Practitioner), says:

In the treatment of pyrosis, or water-brash, the antacids are to be resorted to, particularly the saccharated solution of lime-water and milk—

| | | |
|---|---|---|
| R.—Liquoris Calcis Saccharati, | - | fl. dr. i–iv. |
| Lactis, ad., | - - - - - | fl. oz. iv.—M. |

It may be well to remember that the addition of fifteen grains of bicarbonate of soda to the quart of milk not only prevents it from turning sour, but renders it more digestible.—*New Remedies.*

STRAMONIUM SMOKING.—A. Hirschberg states that when cigarettes of stramonium are smoked for asthma, there is no daturin whatever received in the system, but that the active principles generated are pyridin alkaloids.—*Zeitschrift des Allgem. Oester. Apothek.-Vereins,* June 10, 1872.

Syrupus Cubebæ. — C. L. Mitchell (American Journal of Pharmacy) proposes the following formulæ for the preparation of a syrup of cubebs which has been found an elegant as well as efficacious remedy in diseases of the throat and lungs:

| | |
|---|---|
| Fld. Ext. Cubebs, - - - - - | fl. oz. ij. |
| Carb. Magnesia, - - - - - - | fl. oz. ss. |
| Sugar Powd., - - - - - - | oz. xij. |
| Orange-flower Water, - - - - - | fl. oz. ij. |
| Water, - - - - - - - - | q. s. |
| Ess. Oil Almonds, - - - - - | gtt. j. |

Rub up the fluid extract with the carbonate of magnesia and then add oz. ij. of the powdered sugar in small portions. When thoroughly mixed add gradually, first the orange-flower water, and then fl. oz. vij. water, constantly triturating the mixture until the sugar is dissolved. Filter and add q. s. water through the filter to the amount of f. oz. xj., in which dissolve the balance of the sugar without heat. Add the oil of almonds put in a little alcohol, and again filter, adding, if necessary, q. s. water through the filter, to measure 1 pt.

The dose of this syrup is f. dr. j–iv., and it may be given in even larger doses if desired. It may also be made by using the officinal oleoresin in the proper proportion in place of the fluid extract. —*New Remedies.*

Collodion in Herpes Zoster.—Dr. Wesley M. Carpenter, of Erieville, Madison county, writes:

"I would call the attention of the profession to the use of collodion in this disease. It may have been noticed before this, but I have not happened to see it if such is the case.

"One of the most distressing symptoms in this disease is the intense stinging, burning, superficial pain which often accompanies the eruption. I have reference to the so-called 'characteristic pain,' which may continue a long time after the disappearance of the eruption.

"Great relief may be given to this burning pain by the application of collodion to the surface occupied by the vesicles. This may be done with a feather or camel's-hair brush. The collodion forms a pellicle over the surface of the vesicles, which protects them from the air, and renders them less liable to rupture. It may be applied at any stage of the eruption, and in most cases acts like a charm in giving relief to this annoying accompaniment.

"In general, the disease needs no other local treatment."—*Medical Record.*

TOOTHACHE—SYSTEMIC TREATMENT.—Dr. A. L. Cory (Chicago Medical Times), "reports a new remedy for toothache. Being called upon immediately after the fire, to extract some aching teeth, and not having the necessary implements, and most of the dentists having lost their instruments, he prescribed 20 grains of hydrate of chloral, to lessen the pain and procure rest until he could obtain the necessary forceps. To his astonishment, in every case, the ache not only promptly vanished, but failed to return. The Doctor says he has now tried this plan in about twenty cases with quite uniform success."

Various anodynes will answer the same purpose. Among others, iodoform in one grain doses, is a very efficient remedy for dental and facial neuralgia.—*Medical Cosmos.*

PROF. GROSS' TREATMENT FOR GOITRE.—Treatment will consist in stimulating the absorbent vessels, although the application of agents of too stimulating a character must be avoided, otherwise irritation will be produced, and the mass will be enlarged instead of diminished. The neck will be thoroughly washed at least once in twenty-four hours, with hot water and soap, and immediately afterwards a portion of the following ointment will be applied to the surface of the tumor and well rubbed in—

R.—Ung. Hydrarg. Biniodid, - - dr. j.
Cerat Simp., - - - - - dr. vj.—M.

The patient will take internally the *Liquor iodinii compositus*, gtt. viij., in sweetened water, three times daily.

A piece of thin flannel and oiled silk will be worn around the neck. The diet will be regulated and all red meats avoided. Six grains of blue-mass in combination with a grain of ipecac will be given now and then at bed-time to regulate the secretions.—*N. Y. Medical Journal.*

DR. SIGERSON, an eminent European savant, has found in the air exhaled from the lungs of tea-drinkers a large number of microscopic globules of a poisonous narcotic oil, which explains, he says, why tea makes nervous people coughy. He might have added, according to another high authority, consumptive, for it is claimed that weak people who indulge regularly in this beverage ultimately weaken their lungs, be they ever so strong at first.

HINT FOR SEA-BATHERS.—At Trouville, one of the most fashionable of French sea-side resorts, it is usual to provide warm foot-baths for the bathers when they leave the water. This is said to prevent the headache, from which many persons suffer.

A Diagnostic Sign of Extra-capsular Fractures of the Neck of the Femur.—In a recent monograph upon the subject, M. Jankerguistel (The Boston Medical and Surgical Journal, May 23; from L'Union Medicale) arrives at the following conclusions:

1. Increase in the size of the great trochanter is an accurate and constant sign of extra-capsular fractures of the neck of the femur.
2. The presence of this sign, once ascertained, enables the surgeon to dispense with all those manipulations, often dangerous, always painful, which are necessary to determine crepitation or abnormal mobility.
3. The study of the sign alluded to permits accuracy of diagnosis, without rendering the prognosis more grave, or without compromising a cure.

The Sulphovinate of Soda.—A Parisian pharmacist, M. S. Linvousin, describes the preparation of this salt in the Bulletin de Therapeutique, for March 30th, and recommends it as a mild and efficient saline purgative, with a feeble and rather pleasant taste, free from any tendency to griping, leaving no consecutive constipation, and acting in relatively small doses (25 grammes for adults, 10 grammes for children). He considers it superior to any of the magnesian purgatives, as these, when much used, tend to produce vesical calculi of ammonio-magnesian phosphates. The salt may be also named the ethylsulphate of soda.—*Medical and Surgical Reporter.*

The Uvula.—Dr. Noble Smith condems, in the British Medical Journal, the practice of snipping the uvula, and advocates its complete removal in cases where any operative procedure is called for. He relates two cases simulating consumption, which were at once cured by the removal of the elongated uvula; and says that in merely snipping the organ grows again, and no good results. On the other hand, Sir G. D. Gibb argues, in the Lancet, that the uvula has important functions in deglutition and vocalization, and that its true muscular end does not often become elongated, but only the membrane, and perhaps adipose tissue; consequently, that snipping this part and leaving the muscular fibres intact is quite sufficient, and that no inconvenience arises from this practice.—*The Doctor.*

Dr. W. E. Whitehead considers the rind of bacon the best possible article that can be used for anointing purposes in scarlet-fever.

Seneca in Bronchitis.—Dr. Thomas Barrow (Medical and Surgical Reporter) remarks: Syrup seneca, with its acridness partially obtunded by ant. et potass. tart., has to my knowledge been effective in the permanent cure of hundreds of cases of bronchitis. I have never observed any case in which antimonial wine or mist. glycyr. comp. appeared to produce any alterative effect whatever upon the bronchi. They may be beneficially employed in the first stage of bronchitis; but they can only palliate a cough of long continuance.

Such correctives, however, are exceedingly valuable when employed to render both the syrup and the decoction of seneca less disagreeable to the patients who may have occasion to use either of them.

Ointment for Hernicrania and Neuralgia.—

| | | |
|---|---|---|
| R.—Pure Chloroform, | - - - | dr iii. |
| Cyanide of Potassium, | - - - | dr iis. |
| Axunge, | - - - - - | oz ii. |

Add a sufficient quantity of white wax to make an ointment. Apply to the affected parts frequently. M. C.

Chelonin in Diseases of the Skin.—As a remedy of much value in diseases of the skin, chelonin has for a long time been used quite successfully, both internally and externally.

Cancrum Oris.—

| | | |
|---|---|---|
| R.—Cupri Sulphas, | - - - - | dr ss. |
| Pulv. Cinchona, | - - - - | dr i. |
| Aqua, | - - - - - - | oz i.—M. |

Apply with a camel's hair brush, three times a day.

I have tried this treatment for twenty years, never having it fail in my hands. J. B.

Gelseminum.—In dysentery, combined with opium and rhubarb, have been found sufficient to relieve the gravest forms of the disease. In gonorrhœa, gelseminum is one of our best remedies, especially where the inflammation is of a high grade.

Gelseminum.—In ophthalmia, it is one of our most reliable internal remedies, relieving the congestion and controlling the inflammatory excitement promptly.

Vermifuge.—Two grains of chelonin in combination with three grains of calomel will be found a very efficient remedy for this purpose.

Cause of "Liver Complaint."—Dr. J. E. Spencer believes the most probable cause is the habitual use of swine's flesh. He is strongly inclined to the opinion that the effete particles contained in all greasy or fat pork are lodged in the liver, for it is well known the venous or black blood from all parts of the body charged with impurities, waste matter, and effete particles, passes to the lungs for purification.

Scutellarin.—In chorea, one grain combined with one or two of carbonate of iron, every two or three hours, baths, friction, and well regulated diet, most promptly relieves; and where it is judiciously administered, results in a permanent cure.

Scullcap.—Dr. Cleveland, of Vermont, speaks in strong terms of its efficacy. He also speaks favorably of its concentrated principle, scutellarine, and finds very happy effects from it in quieting nervous disorders. He prefers this plant to all other nervines or anti-spasmodics, in cases where you can wait for its effect.

Tannic Acid in Measles.—In a recent epidemic, even at the beginning of the attack, there was colliquative diarrhœa and a very distressing, harassing cough. For the diarrhœa, I gave tannic acid, and it relieved both that and the cough in a surprising manner. M.

Tic Doloreux.—Prof. Cleveland writes: I have found more relief follow the use of an infusion of the flowers of the arnica montana, in which was dissolved the cyanuret of potassa, both internally and externally. The tincture of belladonna and origanum, applied externally, has given almost instantaneous relief.

A Good Counter Irritant.—

R.—Croton Oil, - - - dr ii.
Spirits of Camphor, - - oz ii.
Alcohol, - - - oz iii.
Spirits Turpentine, - - oz i.—M.

S. Apply freely to the parts. M.

Calumba in Vomiting.—Some writer has said fresh powdered calumba has always, in his hands, proved a most efficacious remedy in nervous, spasmodic or atonic vomiting, as in that of pregnant women. He gives fifteen grains with a few spoonsful of wine, an hour before meals, three times a day.

